# Aging and word predictability during reading: Evidence from eye movements and fixation-related potentials

**DOI:** 10.3758/s13414-024-02981-9

**Published:** 2025-01-28

**Authors:** Ascensión Pagán, Federica Degno, Sara V. Milledge, Richard D. Kirkden, Sarah J. White, Simon P. Liversedge, Kevin B. Paterson

**Affiliations:** 1 School of Psychology and Vision Sciences, George Davies Centre for Medicine, University of Leicester, University Road, Leicester, LE1 7RH UK; 2 Department of Psychology, Bournemouth University, Poole, UK; 3 School of Psychology, University of Central Lancashire, Preston, UK

**Keywords:** Cognitive aging, Word predictability, Context effects, Eye movements, Fixated-related potentials, Reading

## Abstract

The use of context to facilitate the processing of words is recognized as a hallmark of skilled reading. This capability is also hypothesized to change with older age because of cognitive changes across the lifespan. However, research investigating this issue using eye movements or event-related potentials (ERPs) has produced conflicting findings. Specifically, whereas eye-movement studies report larger context effects for older than younger adults, ERP findings suggest that context effects are diminished or delayed for older readers. Crucially, these contrary findings may reflect methodological differences, including use of unnatural sentence displays in ERP research. To address these limitations, we used a coregistration technique to record eye movements (EMs) and fixation-related potentials (FRPs) simultaneously while 44 young adults (18–30 years) and 30 older adults (65+ years) read sentences containing a target word that was strongly or weakly predicted by prior context. Eye-movement analyses were conducted over all data (full EM dataset) and only data matching FRPs. FRPs were analysed to capture early and later components 70–900 ms following fixation-onset on target words. Both eye-movement datasets and early FRPs showed main effects of age group and context, while the full EM dataset and later FRPs revealed larger context effects for older adults. We argue that, by using coregistration methods to address limitations of earlier ERP research, our experiment provides compelling complementary evidence from eye movements and FRPs that older adults rely more on context to integrate words during reading.

**Supplementary information:**

The online version contains supplementary material available at 10.3758/s13414-024-02981-9.

The predictability of a word from its prior sentence context has a major influence on how efficiently that word can be recognised and integrated as part of the reader’s understanding of a sentence (e.g., Rayner, 1998, 2009). Moreover, this use of context is central to the dominant cognitive models of reading (e.g., E-Z Reader, Reichle et al., 1998, 2003; SWIFT, Engbert et al., 2005; OB1-reader, Snell et al., 2018; Chinese Reading Model, Li & Pollatsek, 2020; SEAM, Rabe et al., 2023), all of which incorporate contextual predictability as a key linguistic influence on mechanisms of word identification and eye-movement control (and for broader discussion of the role of linguistic prediction in language processing, see, e.g., Kuperberg & Jaeger, 2016; Pickering & Gambi, 2018).

Evidence for this central role of context comes primarily from studies with college-aged participants, employing methods sensitive to the incremental processing of words. Using behavioral measures of eye movements, Ehrlich and Rayner (1981) showed that college-aged readers spend less time fixating words that are more predictable from the prior context. Moreover, readers are more likely to skip past more predictable words without fixating them. This suggests that contextual knowledge can help readers to process upcoming words parafoveally, so that these words might be recognized without being fixated directly (and for a detailed review of eye-movement research on contextual predictability effects, see Staub, 2015). At the same time, electroencephalographic (EEG) studies using event-related potentials (ERPs) within the same age group show robust effects of contextual predictability on the N400 (a negative-deflected component of the ERP waveform observed 300–500 ms poststimulus onset, peaking around 400 ms), such that the peak amplitude of this component is attenuated for more predictable words ( Kutas & Hillyard, 1984; and for a review of relevant ERP studies, see Kutas & Federmeier, 2011).

According to one view, readers use contextual knowledge predictively, to preactivate either specific lexical items or features of a word’s representation (for a review, see DeLong et al., 2014). This is assumed to allow readers to initiate at least some linguistic processing ahead of time, before encountering the word (Kuperberg & Jaeger, 2016; Pickering & Gambi, 2018). By contrast, an alternative account holds that effects of word predictability are observed only once a word is encountered, influencing how easily it can be integrated as part of the reader’s current understanding of the text, without requiring preactivation of linguistic information (Kintsch & Van Dijk, 1978; McKoon & Ratcliff, 1992; see also Ferreira & Chantavarin, 2018; Luke & Christianson, 2016). Many eye-movement and ERP studies, including those relevant to the present research, cannot decisively distinguish between effects that might be attributable to prediction rather than integration. This is because effects must be observed before a word is encountered for them to be unambiguously attributed to prediction (Pickering & Gambi, 2018). By comparison, studies relevant to the present research typically report effects for words once they are encountered, by examining fixation times or the N400 amplitudes elicited by these words. Consequently, even though findings from these studies often are attributed to predictive processes, they could reflect later processing that occurs during integration. Note that this issue persists even in eye-movement studies that examine context effects on parafoveal processing (e.g., Choi et al., 2017), including effects for word-skipping probabilities. In these studies, readers obtain preview information about an upcoming word, so that any context effects that might be attributable to prediction may also reflect the ease of integrating this previewed information with prior context.

With the current investigation, we focus on whether context effects on the processing of words remain consistent or undergo change across the adult lifespan. The motivation for examining aging effects stems from the observation that, while eye movements and N400 amplitudes provide complementary evidence of context effects for college-aged readers, for older readers there is a divergence in effects in these two measures that remains to be resolved. Resolving this discrepancy is crucial for understanding how cognitive aging influences the reading process. Substantial evidence suggests that even cognitively healthy older individuals exhibit slower and impaired reading performance compared with younger adults. Some accounts attribute these aging effects to cognitive changes impacting on the use of context (for reviews, see Gordon et al., 2015; Leinenger & Rayner, 2017; Paterson et al., 2020; Payne & Silcox, 2019). However, cognitive changes associated with healthy aging could either be detrimental or beneficial to the use of context. On one hand, reduced cognitive processing speed, reductions in working memory capacity, and reduced attentional and executive control in older adulthood all might affect the retention of contextual information needed for predictive processing (e.g., Foos, 1989; Salthouse & Babcock, 1991). On the other hand, the accumulation of vocabulary in semantic memory over a lifetime of reading experience (Ben-David et al., 2015) might facilitate such processes in older readers. This is particularly relevant as existing semantic representations, often associated with crystallized intelligence, appear to remain intact in older age (Stuart-Hamilton, 2012). Accordingly, given the uncertainty surrounding the potential influence of aging on the use of context, it is crucial to establish whether and how context effects differ for older compared with younger adults. It is noteworthy, however, that studies of eye movements and N400 amplitudes, on which our work is grounded, have employed methods that do not differentiate between the effects of prediction and integration, as previously explained. Therefore, while we remain interested in the temporal dynamics of context effects in reading, we focus on whether these effects manifest early during the processing of words—potentially influencing processes associated with lexical identification—or later during processing, where they are more likely to be unambiguously associated with integration.

A prominent hypothesis arising from eye-movement research which is relevant to understanding how context effects might change with age holds that older individuals compensate for a decline in text processing speed by adopting a more “risky” reading strategy (Rayner et al., 2006). This strategy is argued to involve an increased reliance on context to infer the next word in a sentence, leading to more frequent word skipping and, consequently, faster reading. However, this change in strategy also increases the risk of the reader making an incorrect guess about the next word, potentially raising the likelihood of older readers making a regressive eye movement to re-read text to correct those errors. Rayner et al. (2006) proposed that this context-driven word-skipping effect in older readers might be attributed to an increased use of predictive processing, although note that this does not align with current conceptualizations of linguistic prediction (Pickering & Gambi, 2018). Whether older adults exhibit this “risky” reading behavior has nonetheless been a significant issue for eye-movement research on aging effects on reading (see Paterson et al., 2020) and computational modelling of these effects (McGowan & Reichle, 2018), while recent meta-analysis (Zhang et al., 2022; see also Moreno et al., 2019) has shed fresh light on these behaviors.

The meta-analysis by Zhang et al. (2022) confirmed that older adults read more slowly, characterized by longer fixations on words and increased regressions. Interestingly, it also showed that older adults produce higher word-skipping probabilities, although these effects appear to be restricted to studies in alphabetic scripts like English and are not observed in Chinese. This suggests that age-related differences in word skipping may manifest in certain orthographies but not others. Importantly, the meta-analysis provided no evidence to support Rayner et al.’s (2006) hypothesis that a word’s contextual predictability more strongly influences word-skipping probabilities for older readers. However, it did reveal an impact of word predictability on the time older readers spend fixating words. This is especially evident in Chinese studies, where older adults consistently exhibit larger context effects in fixation times compared with younger adults (e.g., Liu et al., 2020; Zhao et al., 2019, 2021). Findings from alphabetic languages, like English, are more variable. For instance, while Rayner et al. (2006) obtained larger word-predictability effects for younger readers in early measures of processing (i.e., first-fixation durations), several studies show larger effects for older readers, but primarily in measures of later processing (Cheimariou et al., 2021; Choi et al., 2017; Steen-Baker et al., 2017; Veldre et al., 2022). Overall, the meta-analysis suggests this age difference in the processing of high- compared with low-predictability words represents an integration effect, especially as differences are not observed in word skipping, as might be expected if they were a result of predictive processing. Moreover, this effect is seen most consistently in Chinese, highlighting the need for research to clarify effects across different orthographies. Crucially, the variability in effects across studies in alphabetic scripts may stem in part from limitations in statistical power. To address this, the present study employed a large sample size and a large stimulus set to ensure a well-powered investigation.

A parallel line of research on the neural correlates of language processing presents a contrasting perspective. Experiments using event-related potentials (ERPs) suggest that, in comparison with young adult readers, older adults derive lesser benefit from the contextual predictability of words (for a review, see Payne & Silcox, 2019). In these studies, a participant’s electroencephalogram (EEG) is recorded from scalp electrodes while they read sentences, typically presented using a rapid serial visual presentation (RSVP) technique. This technique displays words in each sentence sequentially, one at a time, at a fixed rate (usually 200–300 ms with an interstimulus interval of 300–500 ms) at a central screen location, to minimize potential contamination of the EEG recording by eye movements (note that this is a standard procedure in ERP research; see Kutas & Federmeier, 2011).

The EEG signal is time locked to the display onset of specific target words, usually positioned at the end of a sentence. Subsequently, this signal is averaged to generate the ERP waveform, marked by positive and negative charges that produce a series of peaks and troughs thought to be associated with aspects of processing. Numerous ERP studies have explored age differences in the N400 onset latency and amplitude in response to words varying in predictability (Cameli & Phillips, 2000; Dave et al., 2018; DeLong et al., 2012; Federmeier & Kutas, 2005, 2019; Hamberger et al., 1995; la Roi et al., 2020; Payne & Federmeier, 2017; Wlotko & Federmeier, 2012a, b; Wlotko et al., 2012). Generally, the N400 effect is smaller and with a delayed onset for older adults relative to young adults, or even absent in older adults while present in young adults (Cameli & Phillips, 2000). As the N400 component is believed to index the ease of semantic integration with the prior context, with larger amplitudes reflecting more effortful processing (Wlotko et al., 2010), age difference in N400 effects have been taken to indicate that older adults are less adept at using context (Federmeier & Kutas, 2005; Payne & Federmeier, 2018; Wlotko & Federmeier, 2012a, b; Wlotko et al., 2012; see Payne & Silcox, 2019).

While N400 amplitudes in these experiments may not distinguish between effects of prediction versus integration (see Pickering & Gambi, 2018), researchers argue that ERP effect patterns emerging after the N400 provide insights into reanalysis and integrative processes which may be consistent with readers actively generating predictions earlier during processing (see Federmeier, 2022; Lai et al., 2024). Within the ERP literature, these effects appear to manifest as broad patterns of positivity or negativity sustained approximately 500–900-ms poststimulus onset. Some such effects might reflect a processing cost for incorrect predictions (Federmeier et al., 2007; Thornhill and Van Petten, 2012), requiring reinterpretation of contextual information for the reader to attain a coherent understanding (Coulson & Kutas, 2001; Wlotko & Federmeier, 2012a, b). For instance, late positivity appears to be elicited when readers encounter an unexpected word within a highly constrained context (DeLong et al., 2014; Hubbard & Federmeier, 2021; Kuperberg et al., 2020; Payne & Federmeier, 2017; but see Stone et al., 2022; Thornhill & Van Petten, 2012). This has been attributed to the triggering of revision processes when the prediction for a specific word is violated (e.g., Federmeier et al., 2007; Kuperberg et al., 2020; Ness & Meltzer-Asscher, 2018), or increased conflict between a strong contextual representation and unexpected lexical input (Stone et al., 2022). By comparison, late negativity effects appear to be elicited by expected words in strongly constrained contexts.

Wlotko et al. (2012) observed distinct patterns of post-N400 effects for young and older adults. In this study, the young adults exhibited late positivity for unexpected words in strongly constrained contexts, while older adults displayed late negativity for expected words in such contexts. This late negativity was interpreted as showing that older readers engage in increased reconsideration of the prior context on encountering a strongly predicted word. Wlotko et al. suggested that this may represent a late integration process that compensates for the older adults making less effective use of context to generate lexical predictions earlier in processing. However, subsequent studies have reported similar late positivity effects for both young and older adults (Dave et al., 2018; DeLong et al., 2012), while Cheimariou and Morett (2023) reported late positivity effects for older adults and late negativity effects for young adults in a picture–word matching paradigm. Accordingly, while these late ERP effects may reflect processes of reanalysis and integration, the variation in findings across studies introduces some uncertainty about whether this component can reliably indicate differences in processing costs across age groups.

The conclusion, based on N400 amplitudes, that older readers struggle to use context to support word processing contradicts emerging findings from eye-movement research, which suggest that older adults actually exhibit larger, rather than smaller, context effects. It is therefore crucial to establish why the two paradigms appear to produce inconsistent patterns of aging effects. One potential explanation is the use of unconventional stimulus displays in ERP research. As already noted, many ERP studies used RSVP displays to present words in each sentence sequentially, at a fixed rate, to a central screen location, to minimize eye movements. Reading text in these displays in unlike normal reading, as participants cannot read at their own pace, are unable to make regressions to re-read words, and do not process words across multiple fixations, including by parafoveally processing a word prior to it being fixated. It also seems likely that older adults will find adapting to RSVP paradigms challenging. This is because older readers tend to make multiple fixations on words, exhibit slower lexical processing, and are more prone to making regressions to re-read text (Zhang et al., 2022). However, the standard RSVP paradigm used in ERP research requires brief displays to prevent eye movements, which precludes multiple fixations and does not allow for re-reading. It would be unsurprising, therefore, if older adults found this reading experience difficult and potentially struggle to obtain the semantic information necessary for generating contextual expectations. This task-specific difficulty could explain their smaller N400 predictability effects. It is also important to note that comprehension usually is not assessed in these studies, leaving the extent to which readers fully comprehend sentences unclear. Indeed, one issue not addressed in these experiments is the possibility of imbalances in the comprehension achieved by young and older adults in an RSVP paradigm. Crucially, such imbalances could impact on effects elicited by manipulations of word predictability (and for a comprehensive discussion on the differences in processes of word recognition in RSVP displays compared with natural reading, see Kornrumpf et al., 2016).

One response to this critique of ERP research using RSVP displays might be to consider whether similar effects are obtained in speech comprehension, where it is ecologically valid for participants to receive brief exposures to successive words in a sentence without the opportunity to revisit earlier words in the sequence. It is unclear, however, whether similar effects of word predictability are actually obtained in this literature, as many studies investigate more basic priming or word association effects (e.g., Ford et al., 1996; Kutas & Iragui, 1998; Tiedt et al., 2020; Woodward et al., 1993). Moreover, few studies have investigated age differences in sentence context effects, while those that have produce variable findings. For example, whereas Broderick et al. (2021) obtained robust N400 effects of word predictability (using a surprisal measure; e.g., Levy, 2008) that were delayed for older adults, the same study showed that N400 amplitudes for contextually anomalous versus congruent words were reduced for older compared to younger adults (see also Federmeier et al., 2003). Moreover, even if it were the case that speech comprehension and reading studies produced similar patterns of predictability effects, this would not invalidate concerns regarding the use of unnatural display methods in reading tasks that might disadvantage the performance of older participants.

Given these concerns, it is noteworthy that two ERP reading studies that departed from the standard RSVP paradigm produced different outcomes (Hamberger et al., 1995; Payne & Federmeier, 2017). One such study by Payne and Federmeier (2017) is particularly interesting due to its use of an RSVP with flanker paradigm (also explored by Stites et al., 2017). In this paradigm, participants focus centrally while sentences progressively appear across their central vision in a sequence of three-word displays. The middle word always aligns with the participant’s gaze, while the other words are presented to the left and right of this location. This approach ensured that, over the sequence of each three-word display, participants viewed the target words at successive screen locations: first to the right of fixated word, then as the fixated word, and finally to the left of the fixated word. This approach mimics the sequential parafoveal, foveal, and postfoveal processing of words that occurs in natural reading (for languages, like English, that are read from left to right); albeit, in this paradigm the reader views text passively without making eye movements (see Kornrumpf et al., 2016). Following this approach, Payne and Federmeier found graded sensitivity to contextual information presented in parafoveal vision in both young and older participants. More strikingly, though, they obtained larger, not smaller, N400 predictability effects for older adults for target words that appeared in the central location.

Based on these observations, there is a compelling argument for employing more naturalistic paradigms to investigate age differences in the use of contextual information during reading. Coregistration methods, which concurrently record eye movements and EEG during natural reading, are a well-suited approach for this purpose. This paradigm offers the advantage, compared to standard ERP techniques, of allowing experimenters to assess a continuous record of brain activity over time as participants read normally by making saccadic eye movements (for comprehensive overviews, see Degno & Liversedge, 2020; Degno et al., 2021; Dimigen et al., 2011; Himmelstoss et al., 2020). This method is increasingly being used to investigate the neural correlates of language processing during natural reading (e.g., Antúnez et al., 2022; Baccino & Manunta, 2005; Degno et al., 2019a, b; Dimigen et al., 2012; Henderson et al., 2013; Hutzler et al., 2013; Loberg et al., 2018, 2019; López-Peréz et al., 2016; Metzner et al., 2015, 2017; Milligan et al., 2023; Mirault et al., 2020; Niefind & Dimigen, 2016), including to investigate effects of word predictability (Burnsky et al., 2023; Dimigen et al., 2011; Kretzschmar et al., 2015).

The methodology involves time-locking the EEG signal to the onset of the reader’s first fixation on a target word in a sentence, producing a fixation-related potential (FRP) when averaged across multiple trials. The FRP waveform resembles that produced by ERPs, suggesting that it may index the same underlying neural processes. Studies of word predictability have identified a likely FRP analog of the N400 as a negative deflection within a window around 300–500 ms postfixation onset (Dimigen et al., 2011), with Kretzschmar et al. (2015) describing this as peaking around 300 ms poststimulus onset. A noteworthy finding from these studies is that a word’s cloze predictability modulates the amplitude of this component, indicating its sensitivity to a word’s fit within the sentence context. However, FRP studies to date have focused on college-aged readers and so are not informative about age-related changes in the effects of word predictability for this N400-like component. It will therefore be crucial to establish whether young and older adult readers exhibit differences in the onset latency and amplitude of FRP components that index word-predictability effects, similar to the aging patterns reported in standard ERP research.

In the present study, we acquired coregistered recordings of eye movements and EEG from both young (18–30 years) and older (65+ years) adults while they read sentences normally. These sentences contained a target word at a central position within the sentence, with the target word having either high or low predictability based on the preceding sentence context, as determined using the cloze test (Taylor, 1953). Notably, this is the first FRP study with older adult readers and the first to investigate age differences in the effects of word predictability using this methodology.

Care was taken to match the target words for letter length and lexical frequency. We purposely selected reasonably long target and pretarget words (between 5 and 7 letters) to minimize the likelihood of participants skipping target words, thereby maximizing the number of trials available for FRP analysis. This also helped to mitigate age-group imbalances in fixation probabilities, as older readers tend to skip words more frequently (Zhang et al., 2022). In reporting this experiment, we present two analyses of the eye-movement data: one based on the “full EM dataset,” including all data retained postconventional eye-movement screening, and another using a “reduced EM dataset,” further filtered to include only trials suitable for computing FRPs. The latter dataset included the removal of trials where the target word was skipped. These two datasets allowed us to test hypotheses regarding age differences in effects of word predictability on readers’ eye movements. Before testing these hypotheses, we conducted an analysis of sentence-level eye movements to ensure our participant groups exhibited standard age differences in eye-movement behavior. Specifically, we assessed whether, compared with young adults, the older adults read more slowly, made more and longer fixations, and more regressions (see Zhang et al., 2022). Note, however, that we did not anticipate group differences in word-skipping probabilities due to our efforts to minimize this behavior.

Target-word analyses were based on a large stimulus set and sample size. Importantly, we refrained from introducing manipulations (e.g., altering parafoveal word spelling, as done by Choi et al., 2017) that might disrupt normal reading behavior. With this well-powered design, we aimed to effectively test age differences in context effects, which would be clearest in the full EM dataset. While we did not anticipate observing effects in word-skipping probabilities, following the insights from Zhang et al.’s (2022) meta-analysis, we predicted that older readers might show heightened sensitivity to a word’s contextual predictability. We therefore expected them to produce larger context effects in fixation times for target words compared with young adults. It was of particular concern to determine whether these age differences in effects, if present, would manifest in measures of early processing, as noted by Choi et al. (2017) in English and Zhao et al. (2019) in Chinese, or emerge later in the eye-movement record. These distinct outcomes are important because they could shed light on whether context differentially influences early processes associated with word identification or later processes related to the integration of words with their sentence context across different adult age groups.

Conducting an analysis of FRPs enabled us to perform hypothesis-testing and exploratory analyses on the effects of age group and word predictability on this EEG measure of brain activity. Additionally, these effects could be directly compared with patterns of eye-movement behavior from the same participants (recorded concurrently), allowing us to assess the similarity of effects in eye movments and FRPs. If comparable effects occurred, we might observe an FRP effect similar to the N400 ERP effect of word predictability (and we might anticipate, approximately, that any such activation might occur between 300–500 ms after fixation onset). We might also anticipate later FRP effects that could reflect differences in the integration of low relative to high predictable words with sentence context. Crucially, while these effects might occur for both age groups, we anticipated that the effects might (1) be greater for young compared with older adults, which would be consistent with existing ERP findings. Alternatively, (2) effects may be greater in older relative to younger adults, consistent with the older adults’ word recognition or integrative processes operating less efficiently than those of younger adults.

The expectations regarding more general differences in FRPs for young versus older adults were less clear, given that this is the first coregistration study to assess aging effects. However, considering the wealth of evidence indicating slowed processing in older adults, one possibility is that we might observe a lag in the onset of FRP effects for older compared with young readers. Finally, at a broader and more exploratory level, the FRP analyses allow us to assess whether activation associated with earlier stages of processing might occur. Such activation might reflect visual and orthographic/phonological processing of written words (for discussion, see Degno & Liversedge, 2020).

## Method

### Ethics statement

The research was approved by the Research Ethics Committee in the School of Psychology and Vision Sciences at the University of Leicester (ID: 18669) and conducted in accordance with the principles of the Declaration of Helsinki.

### Participants

Participants were 44 young adults aged 18–28 years (*M* = 21 years; 30 women) from the University of Leicester, UK, and 30 older adults aged 65–80 years (*M* = 70 years; 25 women) from the local community. All participants were native English speakers who reported no history of reading difficulties or neurological disorders. Participants provided written informed consent and were paid for participating (£10/hour).

Participant characteristics are summarized in Table 1. The young and older adult groups were closely matched for years of formal education, and all participants were screened for normal visual acuity (better than 20/40 in Snellen values), using an Early Treatment Diabetic Retinopathy Study chart (Ferris & Bailey, 1996). The young adults had higher acuity than the older adults, as it is typical (e.g., Elliot et al., 1995). The older adults were screened for nonimpaired cognitive functioning using the Montreal Cognitive Assessment (MoCA; applying the standard exclusion criterion of scores <26/30; Nasreddine, 2005). The short-term memory and vocabulary capabilities of both age groups were assessed using a digit span task (forward and backward digit span) and a vocabulary knowledge subscale from the *Wechsler Adult Intelligence Scale* (WAIS-IV; Wechsler, 1997). The two age groups did not differ in terms of digit span. However, the older adults had higher vocabulary scores than the young adults, as it typical (e.g., Ben-David et al., 2015; Keuleers et al., 2015).

**Table 1:** Summary of participant characteristics

	Young adults	Older adults	*t*	*p*
Age (years)	21 (2)	70 (4)		
Formal education (years)	16 (2)	16 (3)	1.22	0.223
Visual acuity (Snellen values)	20/25	20/32	2.58	<.012
Vocabulary	41 (5)	46 (6)	3.84	<.001
Digit span	21 (4)	21(4)	0.86	.390

*Note*. The standard deviation of the mean is shown in parentheses. Means for Vocabulary and Digit Span subscales represent scores in the tasks and are not estimates of vocabulary size or digit span

### Stimuli and design

**Fig. 1:**
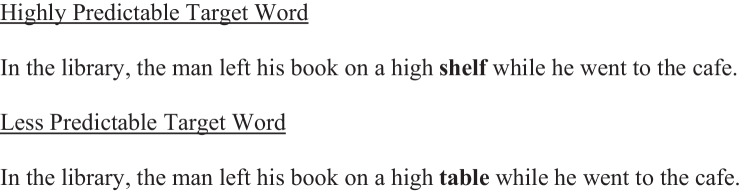
Example sentence stimulus. *Note*. Target words are shown in boldface but were shown normally in the experiment

The stimuli in the reading experiment were 98 sets of sentences which contained an interchangeable 5–7 letter target word that was either highly predictable or less predictable from the prior sentence context. The sentences were 10–22 words long (*M* = 15 words, *SD* = 2 words) and the target word was always located near the middle of each sentence. An example stimulus set is shown in Fig. 1.

Target-word predictability and plausibility were assessed with 20 young and 20 older adults who did not participate in the experiment. Plausibility was examined by asking participants to rate on a scale of 1–7 how plausible they found a given sentence (1 = *highly implausible* to 7 = *highly plausible*). All sentences for both age groups were rated above 4 on average (ratings, though, did differ by age—young adults, *M* = 5.35, *SD* = 1.14; older adults: *M* = 4.65, *SD* = 1.35; *p* = .014, and predictability—high predictability, *M* = 5.75, *SD* = .87; low predictability, *M* = 4.25, *SD* = 1.21; *p* < .001). A cloze task (Taylor, 1953) was used to assess target-word predictability. Participants were provided with the beginning part of a sentence up to but not including the target word and were asked to provide one word to continue the sentence. A target word was considered highly predictability if at least 20% of each age group guessed it to be the next word in the sentence, and of low predictability if 10% or fewer of each age group guessed it to be the next word. The resulting set of target words differed significantly in predictability (high predictability: *M* = 76%, *SD* = 9.16%; low predictability: *M* = .87%, *SD* = 1.09%; *p* < .001), but target-word predictability did not differ between age groups (*p* = .979). The target words were matched for length (high and low predictability: *M* = 5, *SD* = .50), lexical frequency (high predictability: *M* = 4.40, *SD* = .50; low predictability: *M* = 4.30, *SD* = .60), and number of orthographic neighbours (high predictability: *M* = 3.30, *SD* = 3.10; low predictability: *M* = 2.50, *SD* = 2.40), using the SUBTLEX-UK database (van Heuven et al., 2014; all *p* values > .06).

The sentence stimuli were counterbalanced across two lists using a Latin square design. Each included one version of each sentence (i.e., including either the high- or low-predictability target word) and an equal number of sentences containing either a high-predictability or low-predictability target word (i.e., 49 sentences per condition). Each list included an additional 100 filler items that were intermixed with the experimental stimuli. The filler items were sentences from an experiment investigating word frequency effects and so included a target word that had either high or low lexical frequency. While these words were plausible in the sentence context, they were designed to be unpredictable from the prior sentence context. Note that, because the target words in both experiments appeared towards the middle of sentences rather than as a sentence-final word, as is the case in many ERP studies, participants were unlikely to be cued to manipulations of word predictability the present experiment. Participants in each age group were pseudorandomly assigned to lists so that equal numbers of young and the older adults viewed each list. The experiment had a mixed design with the between-participants factor adult age group (young, older) and within-participants factor word predictability (high, low).

### Apparatus

Each participant’s right eye movements were recorded during binocular reading using an EyeLink 1000 Plus eye tracker (SR Research Inc., Ontario, Canada), at a sampling rate of 1000 Hz. Head movements were minimized using forehead and chin rests. Sentence stimuli were presented on a high-definition Benq monitor (1,920 × 1,080 resolution, 144-Hz refresh rate). Sentences were presented in 14-point Courier New font as black text on a light-grey background. At 65-cm viewing distance, three letters subtended approximately 1° of visual angle and so text was of normal size for reading (e.g., Rayner & Pollatsek, 1989).

The EEG signal was recorded using 28 electrodes (ActiCap Snap, Brain Products UK Ltd) located on the scalp following the 10–20 International system. Four monopolar EOG channels recorded the EEG signal associated with eye movements (impedance <10 kΩ). The AFz electrode was used as a ground and the FCz electrode was used as the online reference. The EEG signal was recorded from a BrainAmp DC amplifier (Brain Products UK Ltd) with a 1000-Hz sampling rate, and low-pass filtered online at 250 Hz.

### Procedure

Participants took part individually and were instructed to read sentences from the computer display silently and for comprehension. Prior to the start of the experiment, participants completed the Edinburgh handedness inventory (Oldfield, 1971) to ensure right-handedness. Participants also were tested for visual acuity, the older adults were screened for nonimpaired cognitive function, and all participants completed assessments of short-term memory and vocabulary knowledge. The setup and calibration of the eye tracker was tested for each participant to ensure that their eye gaze could be recorded accurately. Participants who completed these tasks progressed with electrode preparation and the experimental session. At the start of the experiment, a 3-point horizontal calibration of eye gaze was performed across the same horizontal line as each sentence was presented (ensuring .20° or better accuracy for all participants). Calibration accuracy was checked before each trial, and the eye tracker recalibrated as required to maintain high spatial accuracy. Once the eye tracker was calibrated, the participant was first shown three practice items, followed by 10 separate blocks of experimental stimuli and filler items.

At the start of each trial, a fixation cross the same size as a letter space was presented on the left side of the screen. Once the participant fixated this location for 500 ms, a sentence was presented with its first letter replacing the cross. Once the participant finished reading the sentence, they made a saccade to fixate a second cross to the right of the sentence. The sentence then disappeared and was replaced on 40% of trials by a yes/no comprehension question. Participants responded by pressing one of two keys on a Cedrus RB-50 response box. Participants received a short break at the end of each block but were permitted to take a break at any point. The experimental session lasted about 1 hour.

### Data preprocessing

Eye movement and EEG data were processed largely in line with the procedures detailed by Degno et al. (2021). Eye-movement data were parsed using Dataviewer software (SR Research inc.) and a customized script in R. Trials were excluded from fixation time analyses if the target word was skipped or participants blinked while looking at the target word during first-pass reading. Accordingly, only trials in which a first-pass fixation was made on the target word were included in fixation time analyses.

EEG data were preprocessed using EYE-EEG extension (Dimigen et al., 2011) of EEGLAB toolbox (Version 14_1_2b; Delorme & Makeig, 2004) for MATLAB (Version 2018a). Eye movements and the EEG signal were synchronized offline. For this, the stimulus display computer, running SR Research Experiment Builder, sent an onset and offset trigger at the start and the end of each trial to the computer recording the EEG signal and a message to the computer registering eye movements. Accurate synchronization was confirmed by deviations equal or shorter than 1 ms in absolute value (*M* = 0.31 ms, *SD* = 0.05).

EEG data were first filtered using a high-pass filter of 0.1 Hz and then a low-pass filter of 100 Hz to improve the signal–noise ratio and to increase statistical power for detection of effects (e.g., Luck, 2014). For the Independent Component Analysis (ICA), we created FRP training data (Dimigen, 2020) by applying a stricter high-pass filter with a passband edge of 3 Hz (width of transition band: 2 Hz, low cutoff [−6dB]: 2 Hz) with low-pass filtering at 100 Hz. In addition, we cut and mean-centred short 30 ms epochs (−20 to +10 ms from saccade onset) and appended the spike potentials corresponding to 30% of the original training data to remove any residual saccade spike artefacts. We then ran the ICA with the training data by down sampling the training data to 500 Hz to save time and computer resources. Finally, we used the EYE-EEG extension to identify ocular ICs and adopted a variance ratio threshold of 1.2 as oculomotor artefacts.

Once ICA weights were computed and ocular ICs identified, the weighted matrix was applied to the original EEG dataset with a less strict high-pass filtering (0.1 Hz), and ocular ICs were removed (*M* = 3.76, *SD* = 0.96). Finally, a 30-Hz low-pass filter was used to remove high frequencies associated with muscle artefacts and power line noise. The EEG signal was segmented into epochs of 1,150 ms cut around fixation onset (−200 ms to +950 ms). To exclude nonocular artefacts, segments with a peak-to-peak voltage difference greater than 150 µV (in absolute value) in any scalp channel were rejected (*M* = 0.4 segments removed per participant, *SD* = 0.9). Spherical interpolation of a channel was performed when the channel exceeded the threshold for more than 5% of all epochs. EEG segments were then rereferenced against the mean of all scalp electrodes (average reference) and baseline-corrected by subtracting 100 ms preceding the fixation onset on the target word. FRPs were then averaged within and then across participants for analyses. A total of 5,174 observations were available once eye-movement and FRP pre-processing was completed (see Table 2).

**Table 2:** Number and mean (*SD*) of observations per condition for reduced EM dataset

	Young adults		Older adults	
Observations	High predictability	Low predictability	High predictability	Low predictability
Number	1465	1,584	1,041	1,083
Mean (*SD*)	33 (7)	36 (6)	35 (6)	36 (6)

### Eye-movement data analysis

Eye-movement analyses were conducted for two datasets: (a) a full eye-movement dataset and (b) a reduced eye-movement dataset, obtained after FRP preprocessing was completed. For both datasets, eye movements were analysed by generalized linear mixed-effects models using the lme4 package (Bates et al., 2011) in the R statistical programming environment (R Development Core Team, 2019). These analyses were performed using the glmer function. Untransformed data and the gamma family and identity link were used for continuous variables (i.e., eye fixation times) to reduce skew (Lo & Andrews, 2015), and the binomial family was used for dichotomous variables (i.e., word-skipping and regression probabilities). Age group and word predictability were specified as fixed factors using the “contr.sdif” function in the MASS package (Venables & Ripley, 2002). Contrasts were specified as 0.5/−0.5 for effects of age group (young versus older adults) and word predictability (high vs low predictability), such that the intercept corresponded to the grand mean. Participants and items were specified as random factors. A maximal random effects structure (Barr et al., 2013) was used. If the model failed to converge, we increased the number of iterations using the BOBYQA optimizer (Powell, 2009). If the model still failed to converge, we trimmed its random structure until it did converge (by first removing correlations between factors, then interactions). All findings reported here are from models that converged.

Additional analyses were conducted to explore effects for the pretarget and posttarget words. For the pretarget words, we obtained effects of age group but not word predictability. For the posttarget words, we obtained effects of word predictability, age group, and interactions of these effects (particularly in later measures). The findings showed that no effects of word predictability were obtained prior to the readers encountering the target word, while the posttarget word produced typical patterns of spillover effects. These additional analyses are available as supplementary information in our OSF files.

### FRP data analysis

A two-tailed nonparametric cluster-based permutation test (Maris & Oostenveld, 2007) was used for FRP data analysis, using the MATLAB toolbox *FieldTrip* (Oostenveld et al., 2011) to minimise the multiple comparison problem (Groppe et al., 2011; Maris & Oostenveld, 2007). All scalp electrodes and all time points of interest from 70–900 ms were included in the test. For the null distribution, permuted data were generated with 20,000 iterations by randomly assigning the condition labels of each participant’s response averages on each iteration. For each iteration, we computed a dependent-sample *t* statistic for each sample (channel–time pair) for the difference between the conditions (high vs low; main effect of predictability); and an independent-sample *t* statistic for the difference between adult age groups (older vs young; main effect of age group) and for the difference between the conditions in each age group (interaction between age group and predictability). Spatial adjacency was defined using the method “triangulation” of the “ft_prepare_neighbours” function in FieldTrip (Oostenveld, et al., 2011). If the *t* statistic was less than .05 and temporally and spatially adjacent to another point with a significant *t* value, the *t* statistic was assigned to a cluster. For each iteration, we summed *t* values within a cluster and computed the maximum positive and negative cluster-level *t* statistic as an absolute value. For the observed data, we followed the same procedure as the permuted data, but did not randomly assign trials between conditions. Finally, the observed *t* statistic was tested against the null distribution. A *p* value less than .025 was considered statistically significant when this was within the 5% most extreme maximum/minimum cluster-level *t* statistics in the null distribution.

## Results

### Comprehension question accuracy

Accuracy answering the comprehension questions that followed sentences was high for all participants and did not differ across age groups (young adults: *M* = 91%, *SD* = 2.7%; older adults: *M* = 91%, *SD* = 3.7%; *p* > .8), indicating that both age groups understood the sentences well.

### Analysis of full eye-movement dataset

Following standard procedures, fixations more than 80 ms and less than 1,200 ms were included for sentence-level and target-word-level analyses. A total of 7,644 observations were analysed. We ran analyses to examine age-group effects in the following sentence-level measures: sentence reading time, average fixation duration, number of fixations, number of regressions (backwards eye movements in the text), and forward saccade length (the average length, in letters, of progressive eye movements). Table 3 shows mean sentence-level eye movements for the young and older adults and summarizes statistical effects for age-group comparisons.

**Table 3:** Means and statistical effects for sentence-level eye-movement measures

	Young adults	Older adults		
	*M* (*SD*)	*M* (*SD*)	*t*	*p*
Sentence reading time (ms)	6,698 (2409)	8,206 (3761)	574.10	<.001
Average fixation duration (ms)	232 (35)	263 (41)	5.09	<.001
Number of fixations	14 (4)	16 (6)	2.83	.005
Number of regressions	2.6 (2.1)	3.5 (2.3)	3.38	.001
Progressive saccade length (letters)	1.8 (0.7)	1.9 (0.6)	0.20	.839

Compared with the young adults, the older adults had longer sentence reading times, made more and longer fixations, and more regressions. These results are consistent with previously reported adult age differences (see Zhang et al., 2022). We observed no differences in the length of forward saccades, although some previous research has reported that older readers make generally longer forward saccades. Note that the absence of an age difference in the length of forward saccades is inconsistent with the “risky” reading hypothesis (Rayner et al., 2006), as a higher word-skipping rates would likely also result in longer forward saccades on average.

For the target-word analyses, we examined eye-movement measures sensitive to the first-pass processing of target words (i.e., prior to a saccade to the right of this word or a regression to its left), as well as measures of later processing. As first-pass measures, we examined word-skipping probability (probability of the target word not being fixated during first-pass reading), first-fixation duration (the length of the first fixation on the target word during first-pass reading), single-fixation duration (duration of the first-pass fixation time on a target receiving only one first-pass fixation), gaze duration (sum of all first-pass fixations on the target word), and regressions out (probability of a backwards eye movement from the target word following a first-pass fixation on this word). As measures of later processing, we examined regression-path reading time (also known as go-past time, the sum of all fixations from the first fixation on the target word until a fixation to its right, including fixations made following a regression; Liversedge et al., 1998), and total reading time (sum of all fixations on the target word). Table 4 shows means and standard deviations for target word measures and Table 5 summarizes statistical effects.

**Table 4:** Mean target word eye-movement measures for the full and reduced datasets

	SP		FFD		SFD		GD		RO		RPRT		TRT	
	HP	LP	HP	LP	HP	LP	HP	LP	HP	LP	HP	LP	HP	LP
**Full EM dataset**														
Young adult	.27 (.4)	.21 (.4)	226 (66)	250 (80)	226 (64)	248 (76)	237 (79)	266 (95)	8 (3)	10 (3)	257 (108)	298 (145)	263 (121)	311 (155)
Older adult	.23 (.42)	.21 (.41)	274 (81)	301 (90)	272 (83)	296 (94)	288 (95)	324 (124)	14 (3)	15 (4)	333 (170)	392 (215)	328 (159)	419 (231)
**Reduced EM dataset**														
Young adult			227 (66)	249 (77)	227 (65)	249 (77)	237 (79)	266 (96)	4 (2)	6 (2)	253 (102)	298 (142)	278 (138)	331 (179)
Older adult			280 (83)	307 (92)	277 (84)	301 (98)	292 (97)	329 (130)	13 (3)	17 (4)	343 (177)	402 (214)	361 (184)	466 (271)

*Note*. Separate analyses are reported for the full eye-movement dataset and for a reduced eye-movement dataset corresponding to the observations used in the FRP analyses. Standard deviations of the mean are shown in parentheses. For word predictability, HP = high predictability, LP = low predictability. For the reported eye-movement measures, SP = skipping probability, FFD = first-fixation duration, SFD = single-fixation duration, GD = gaze duration, RO = the percentage probability of a first-pass regression-out, RPRT = regression-path reading time, and TRT = total reading time. All measures are in ms except for SP and RO. SP is not reported for the reduced EM dataset as these data were filtered to remove trials on which the target word was skipped

**Table 5:** Summary of statistical effects for eye-movement analyses

		Full EM dataset				Reduced EM dataset			
		Estimate	*SE*	*t/z*	*p*	Estimate	*SE*	*t/z*	*p*
Skipping probabilities	Intercept	−1.38	0.1	−14.33	<0.001				
	Older-Young	−0.16	−0.18	−0.91	0.362				
	LP-HP	−0.25	0.08	3.28	<0.001				
	Older-Young: LP-HP	0.22	0.14	1.54	0.124				
First-fixation duration	Intercept	265.70	3.98	66.68	<.001	268.10	5.64	47.54	<.001
	Older-Young	49.61	5.36	9.25	<.001	51.58	6.69	7.71	<.001
	LP-HP	23.65	2.98	7.94	<.001	23.55	3.85	6.12	<.001
	Older-Young: LP-HP	3.55	3.78	0.94	0.347	2.91	5.73	0.51	0.612
Single-first duration	Intercept	274.43	4.74	57.9	<.001	277.82	5.72	48.54	<.001
	Older-Young	53.82	8.43	6.38	<.001	58.41	6.47	9.03	<.001
	LP-HP	28.80	3.68	7.81	<.001	27.29	4.14	6.60	<.001
	Older-Young: LP-HP	6.56	5.42	1.21	0.226	8.36	5.29	1.58	0.114
Gaze duration	Intercept	283.19	4.22	67.08	<.001	285.53	6.11	46.74	<.001
	Older-Young	56.16	5.68	9.88	<.001	57.80	6.07	9.52	<.001
	LP-HP	32.12	3.68	8.72	<.001	33.37	4.10	8.14	<.001
	Older-Young: LP-HP	8.14	4.74	1.72	0.086	7.49	5.49	1.36	0.173
Regressions–out	Intercept	−2.36	0.11	−21.27	<.001	−5.01	0.58	−8.58	<.001
	Older-Young	0.64	0.19	3.44	<.001	3.02	0.90	3.36	<.001
	LP-HP	0.17	0.10	1.63	0.104	1.17	0.79	1.47	0.142
	Older-Young: LP-HP	−0.10	0.16	−0.64	0.522	−0.12	0.86	−0.14	0.886
Regression–path reading time	Intercept	325.67	3.63	89.6	<.001	331.37	6.46	51.32	<.001
	Older-Young	83.01	4.07	20.38	<.001	91.62	7.85	11.68	<.001
	LP-HP	50.69	3.49	14.53	<.001	52.31	4.50	11.64	<.001
	Older-Young: LP-HP	17.79	4.40	4.05	<.001	10.49	6.86	1.53	0.126
Total reading time	Intercept	337.00	7.79	43.29	<.001				
	Older-Young	86.36	7.26	11.89	<.001				
	LP-HP	72.18	5.67	12.73	<.001				
	Older-Young: LP-HP	41.67	5.79	7.19	<.001				

*Note*. Separate analyses are reported for the full EM dataset and for a reduced EM dataset corresponding to observations used in FRP analyses. Note that skipping probabilities are not reported for the reduced EM dataset as these data was filtered to remove trials on which the target word was skipped. Total reading times also are not reported for the reduced EM dataset the glme model failed to converge. The glme model for all fixation time measures was glmer(depvar ~ Group × Predictability + (1 + Predictability|pp) + (1 + Predictability|stim), control = glmerControl (optimizer = “bobyqa”, optCtrl = list(maxfun = 10000)), data = datafile, family = “Gamma” (link = “identity”)). For fixation probabilities and regression-out, the glme model was glmer(depvar ~ Group × Predictability + (1 +Predictability|pp) + (1 + Predictability|stim), control = glmerControl (optimizer = “bobyqa”, optCtrl = list(maxfun = 10000)), data = datafile, family = binomial)). Note that for the reduced eye-movement dataset, skipping probabilities were not analysed as trials on which words were skipped were excluded from this analysis. In addition, the glme model for total reading time failed to converge and so we do not report its results

Compared with the young adults, the older adults had longer reading times for target words, during both first-pass and later processing, and were more likely to make a first-pass regression from the word (i.e., regressions out). These effects were in line with previously reported age-group differences (see Zhang et al., 2022), providing further evidence that our participant groups were representative of young and older adult groups in previous research.

We also obtained a significant word-predictability effect in all target word eye-movement measures (except regressions out). Fixation times were shorter for the more predictable words during both first-pass and later processing, consistent with previous demonstrations of a processing advantage for more predictable words (e.g., Balota et al., 1985; Bélanger & Rayner, 2013; Drieghe et al., 2005; Kretzschmar et al., 2015; Rayner et al., 2004, 2011; Rayner & Well, 1996; Reichle & Drieghe, 2013).

Crucially, we obtained an interaction between age group and predictability in regression-path reading times and total reading times for the target words. These were due to the older readers producing a larger word-predictability effect (i.e., a larger increase in reading times for the less predictable compared with highly predictable words) compared with the young adults. This interactive pattern is particularly noteworthy as all earlier local measures showed solely main effects. The fact that the interactive pattern emerged in late measures of processing suggests it does not represent an effect of lexical prediction (whereby readers are anticipating the next word in a sentence) but is associated with postlexical, structural, and interpretative aspects of comprehension, including the integration of the target word with its prior sentence context. To be clear, that the predictability effect was larger for the older adults suggests that these readers were less efficient in their postlexical processing (cf. Choi et al., 2017). Note that this effect is inconsistent with the “risky” reading hypothesis (Rayner et al., 2006), which predicts larger context effects for older readers early during processing.

The effects we observed in late measures clearly show age differences in the processing of predictable words. This could be explained in terms of the older readers benefiting more from a word’s high predictability during integration or having greater difficulty integrating less predicted words. Note, however, that the less predictable words were not only unexpected but also disconfirmed a more expected continuation. The effects we observed might, therefore, be explained in terms of the older readers having greater difficulty suppressing an incorrect prediction and/or revising their existing representations of prior sentence context to integrate the unpredicted word. One possibility, therefore, is that older readers are not only less efficient at integrating unpredictable words but also have greater difficulty resolving prediction error. We cannot presently distinguish between these possibilities, so future studies should investigate this issue using methods that are informative about prediction error processing (e.g., Frisson et al., 2017).

### Reduced eye-movement dataset

As noted earlier, we computed eye-movement measures for the observations remaining following FRP preprocessing. When preprocessing the FRP data, we included only those trials on which the target word received at least one first-pass fixation (i.e., was not skipped during first-pass reading). Consequently, the reduced dataset included fewer target word observations than the full data set (i.e., a total of 5,174 observations as compared with 7,644 observations for the full dataset). Accordingly, for completeness, we report only target word eye movements for this reduced dataset, as these include the observations used for the FRP analyses. Tables 3 and 4 report the mean eye-movement data and a summary of the statistical analyses, respectively.

Consistent with analyses for the full dataset, we obtained main effects of age group in all eye-movement measures. As before, this was because, compared to the young adults, the older adults had longer reading times for target words, during both first-pass and later processing, and made more regressions. We also observed main effects of word predictability in all eye-movement measures (except regressions out). Again, this was because reading times, during both first-pass and later processing, were shorter for highly predictable compared with less predictable words. The interaction between age group and word predictability in regression-path reading times and total reading times in the full dataset was not reliable in the reduced dataset, most likely because of reductions in statistical power for the reduced eye-movement dataset.

### Fixated-related potentials

FRP mean amplitudes were time locked to the first first-pass fixation onset on the target words over the time window from 70-900 ms. This time window was chosen to allow us to observe patterns of activation that might reflect early FRP components, such as P1 and N1, which are known to show effects of visual and orthographic/phonological manipulations, and later components, such as N400, the P600, and late frontal positivity/negativity that show effects of lexical, semantic and syntactic manipulations. Table 6 summarizes the statistical differences obtained using cluster-based permutation tests.^1^

**Table 6:** Summary of statistical effects in fixation-related potentials when considering a single time window of analysis, between 70-900 ms after first fixation onset on the target

Effects	Cluster		
		*N*	*p*
Effects			
Group (Older-Young)	Positive	2 (3)	< .008*
	Negative	2 (3)	< .014*
Predictability (Low-High)	Positive	0 (7)	(> .071)
	Negative	1 (6)	< .007*
Group x Predictability	Positive	1 (12)	< .009*
	Negative	4 (8)	< .013*
Contrasts			
Young	Positive	0 (2)	(> .151)
(Low vs High Predictability)			
	Negative	0 (1)	(> .089)
Older	Positive	4 (14)	< .023*
(Low vs High Predictability)			
	Negative	4 (9)	< .020*

*Note*. Significance is when *p* values are < .025. Asterisks show clusters that are significant. In brackets the total number of clusters found, outside the brackets the number of significant clusters found. *N*, Number of clusters found

### Age-group effects

**Fig. 2:**
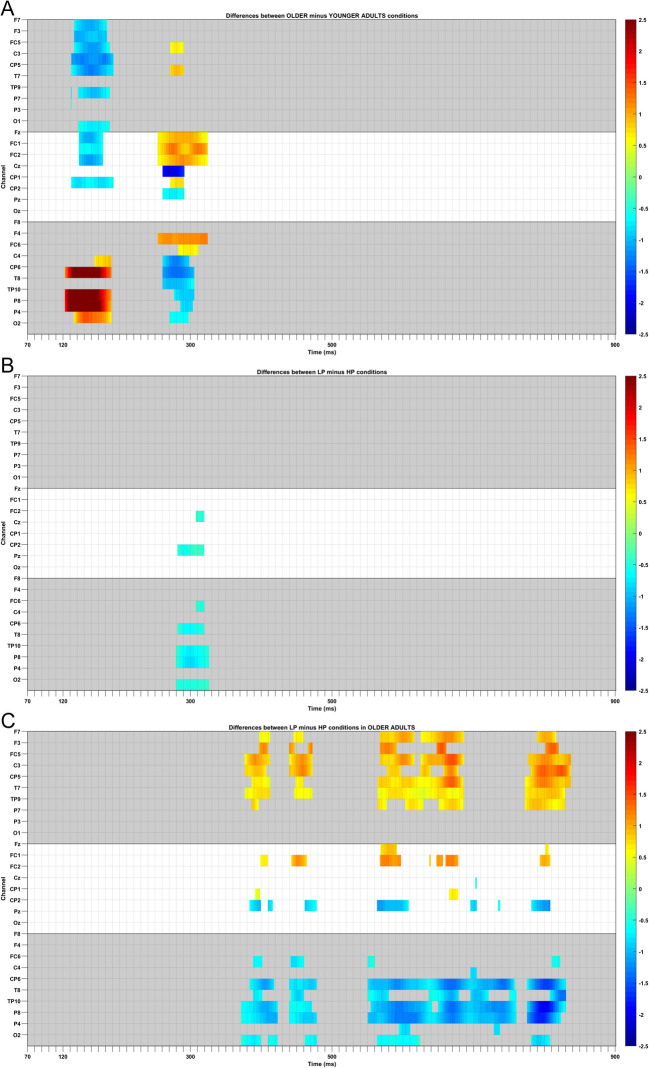
Raster diagrams of significant FRP effects using cluster-based permutation tests. **A** FRP differences between older and younger adults.** B** FRP differences between low predictability (LP) and high predictability (HP) target words.** C** FRP differences between low predictability (LP) and high predictability (HP) target words for older adults. Note that FRP difference for LP and HP for the younger adults are not included as these showed no significant effects. Red and blue rectangles indicate the channel/time point in which one levels of a condition (i.e., older versus younger adults, low predictability [LP] versus high predictability [HP]) is significantly more positive or negative than the second condition. Channels are displayed on the *y*-axis and organized somewhat topographically. Channels within the left hemisphere of the scalp are shown on the top grey rectangle. Midline electrodes are displayed in the middle (unfilled) section, and channels within the right hemisphere are shown on the figure’s bottom grey rectangle. The time from the onset of a fixation on the target words is displayed on the *x*-axis. (Colour figure online)

**Fig. 3:**
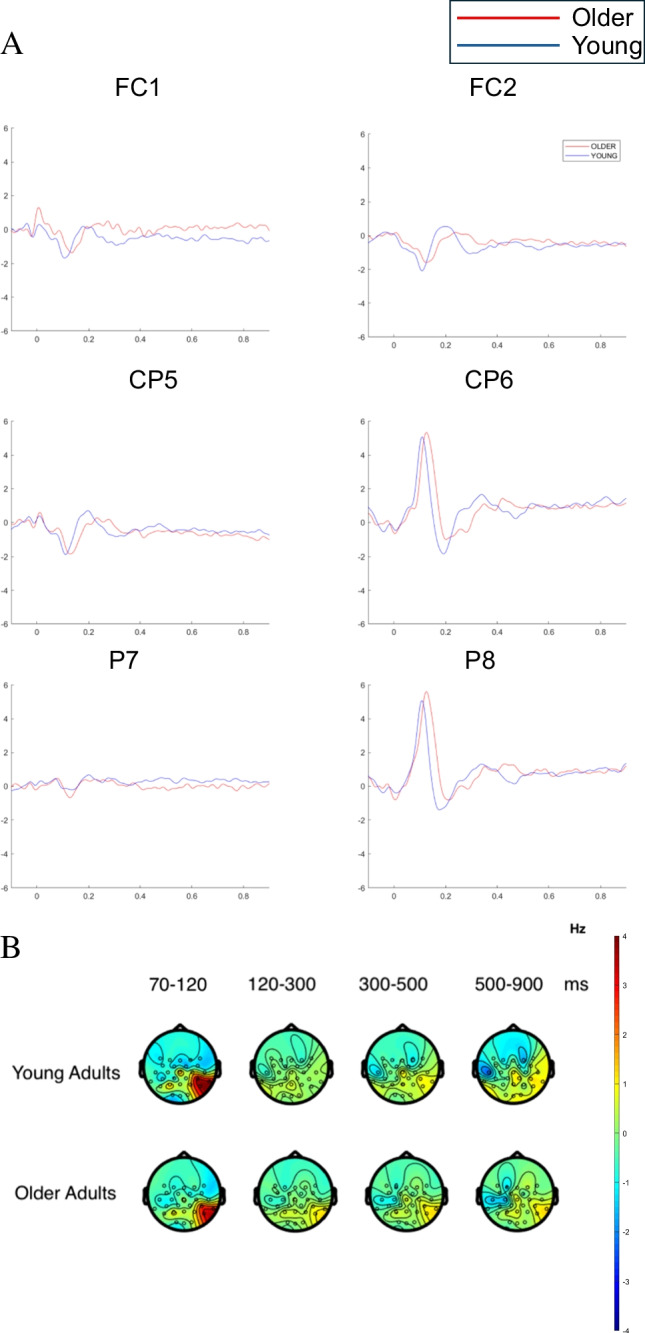
FRPs for the effect of age group. FRPs were time-locked to the onset of the initial first-pass fixation on the target word in each sentence. **A** Grand average FRPs for older and younger adults across six channels: left fronto-central (FC1), right fronto-central (FC2), left centro-parietal (CP5), right centro-parietal (CP6), left parietal (P7), and right parietal (P8). **B** Topographies of average brain activity for older and younger adults across four time windows: 70–120 ms, 120–300 ms, 300–500 ms, and 500–900 ms after the onset of the initial first-pass fixation on the target word in each sentence. (Colour figure online)

We first consider age-group differences. The raster plot in Fig. 2A shows effects of age group according to the cluster-based permutation tests (see Table 6 for statistical results), and Fig. 3 depicts FRPs for age-group effects. Figure 2A shows significant voltage differences between the young and older age groups. As can be seen from the raster plot, there were significant differences in activation between the young and older adults relatively early in our time window. The earliest cluster of positive differences was observed over posterior brain regions; that is, right centro-parietal, temporo-parietal, and parietal brain regions. Likely due to the polarity of the brain activity, the earliest cluster of negative voltage differences between older and younger adults was observed over central and left dipolar regions. A somewhat later cluster of negative voltage differences was maximal over midline and right central and centro-parietal regions, while a cluster of positive voltage differences was largest over midline and right frontal and fronto-central regions.

Considering the waveforms and topographies associated with these clusters (see Fig. 3), it appears that the young adults produced an earlier and more negative N1 component, followed by a more positive P2 or N400-like component compared to the older adults. Accordingly, the presence of two clusters might represent two aspects of processing, with the earliest cluster showing effects of orthographic processing, and the later cluster reflecting phonological or lexical processing of words, as suggested by Degno et al. (2019b). Note that this resembles letter-to-word form processing during the N1/P150 latency and subsequent letter-to-whole-word form processing during the N250 latency described by Grainger and Holcomb (2009). This interpretation is consistent with faster visual encoding of target words by the young adults enabling the rapid initiation of subsequent orthographic, phonological and lexical processes. By comparison, because of their slower visual encoding these subsequent stages of processing may proceed more slowly for older adults.

### Word-predictability effects

**Fig. 4:**
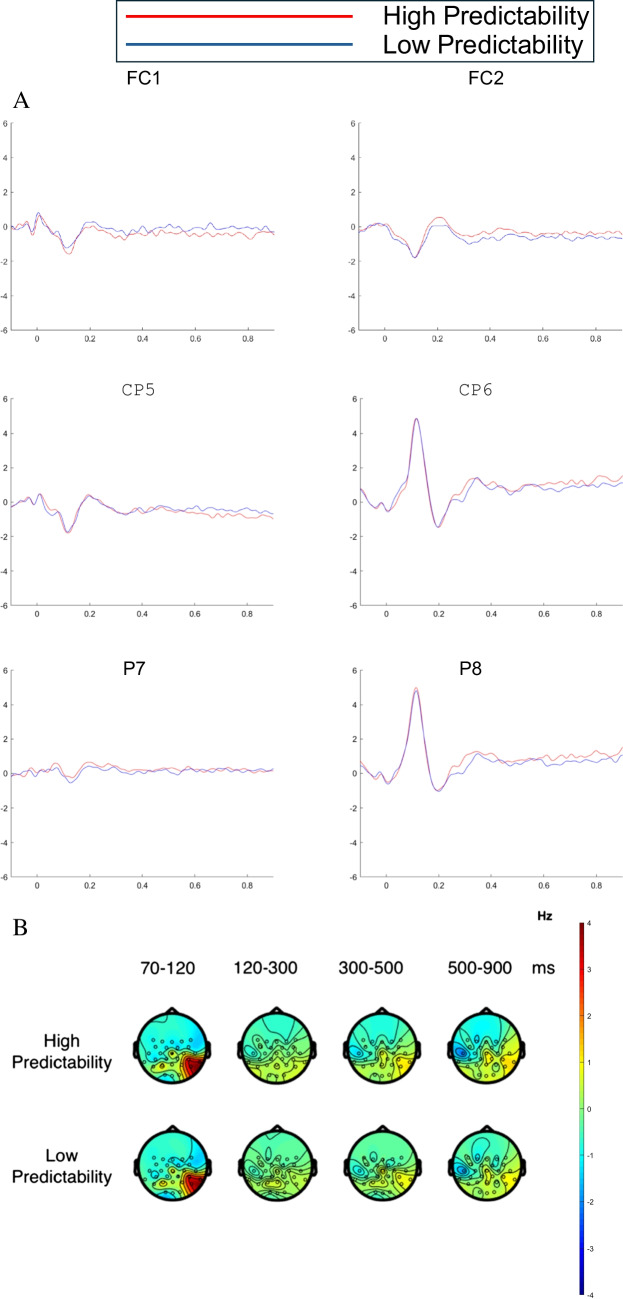
FRPs for the effect of word predictability.FRPs were time-locked to the onset of the initial first-pass fixation on the target word in each sentence.** A** Grand average FRPs for highly predictable and less predictable target words across six channel locations: left fronto-central (FC1), right fronto-central (FC2), left centro-parietal (CP5), right centro-parietal (CP6), left parietal (P7), and right parietal (P8). B Topographies of the average brain activity associated highly predictable and less predictable target words forfourtime windows: 70–120 ms, 120–300 ms, 300–500 ms, and 500–900 ms after the onset of the initial first-pass fixationonthetargetword in each sentence. (Colour figure online)

Next, we consider effects of word predictability. The raster plot in Fig. 2B shows effects of word predictability according to the cluster-based permutation tests, and Fig. 4 depicts FRPs for these effects. The cluster-based permutation tests reveal significant differences between the less predictable and the highly predictability words around 300-ms postfixation onset (see Fig. 2B), where we observed a significant single cluster of negative voltage differences across the two word-predictability conditions. These negative differences were maximal over right occipital, temporo-parietal, parietal, centro-parietal electrodes and right fronto-central electrodes. The waveforms in Fig. 4 suggest that these differences reflect a traditional word-predictability effect, where less predictable words elicit more negative amplitudes than more predictable words. These findings, alongside the eye-movement results, confirm that our manipulation of word predictability was effective and produced detectable effects in both data sets.

The results suggest that following an initial period during which the two types of words were processed differently, later, higher-level stages of processing proceeded similarly. Thus, we consider it important to point to the contrasting nature of the word-predictability effects and the age-group effects we obtained here. Waveforms associated with low and high word predictability appear closely temporally aligned, with only a very short period during which amplitudes associated with the two types of word differ. By contrast, for the age-group effect, the entire waveforms appear to be temporally shifted relative to each other, meaning that components in each waveform are similar, yet those components appear consistently earlier in time for the young adults relative to the counterpart components for the older adults. Thus, to reiterate, the FRP effects associated with the predictability manipulation and those reflecting age-group differences appear to be qualitatively different.

### Age-group × word-predictability effects

**Fig. 5:**
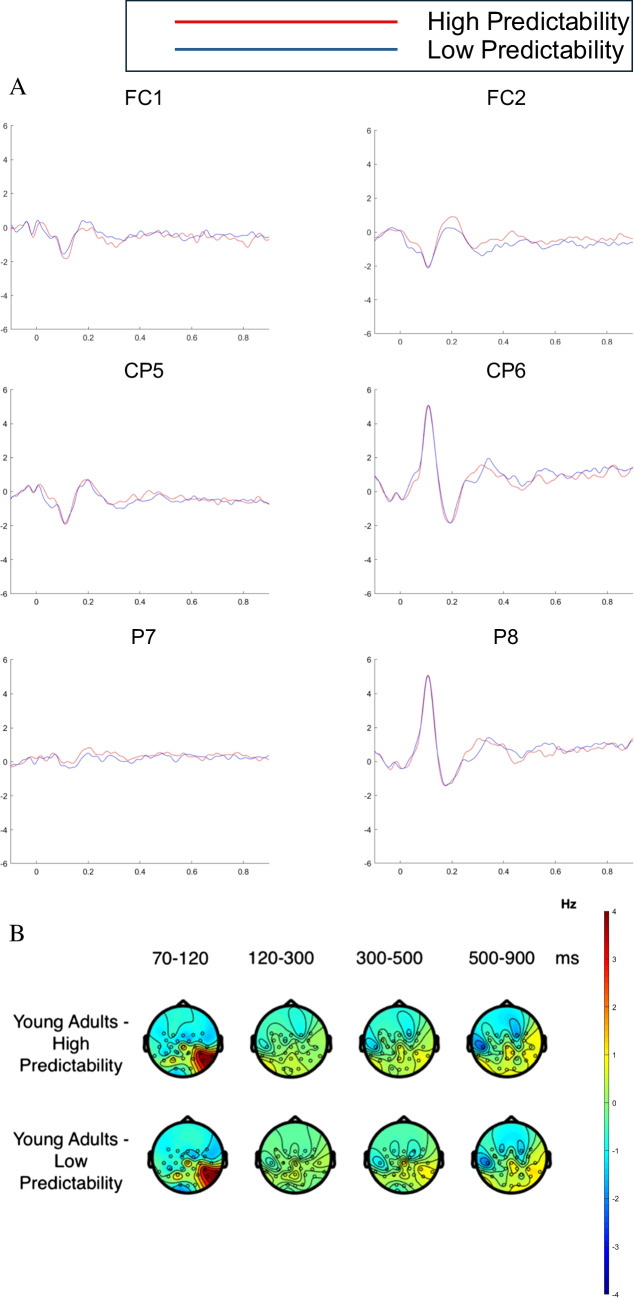
FRPs for the word-predictability effect for young adults. FRPs were time-locked to the onset of the initial first-pass fixation on the target word in each sentence.** A** Grand average FRPs associated with the word-predictability effect (LP versus HP) for young adults across six channel locations: left fronto-central (FC1), right fronto-central (FC2), left centro-parietal (CP5), right centro-parietal (CP6), left parietal (P7), and right parietal (P8) electrodes.** B** Topographies of the average brain activity associated with the word-predictability effect (LP versus HP) for young adults for four time windows: 70–120 ms, 120–300 ms, 300–500 ms, and 500 –900msafter the onset of the initial first-pass fixation on the target word in each sentence. (Colour figure online)

**Fig. 6:**
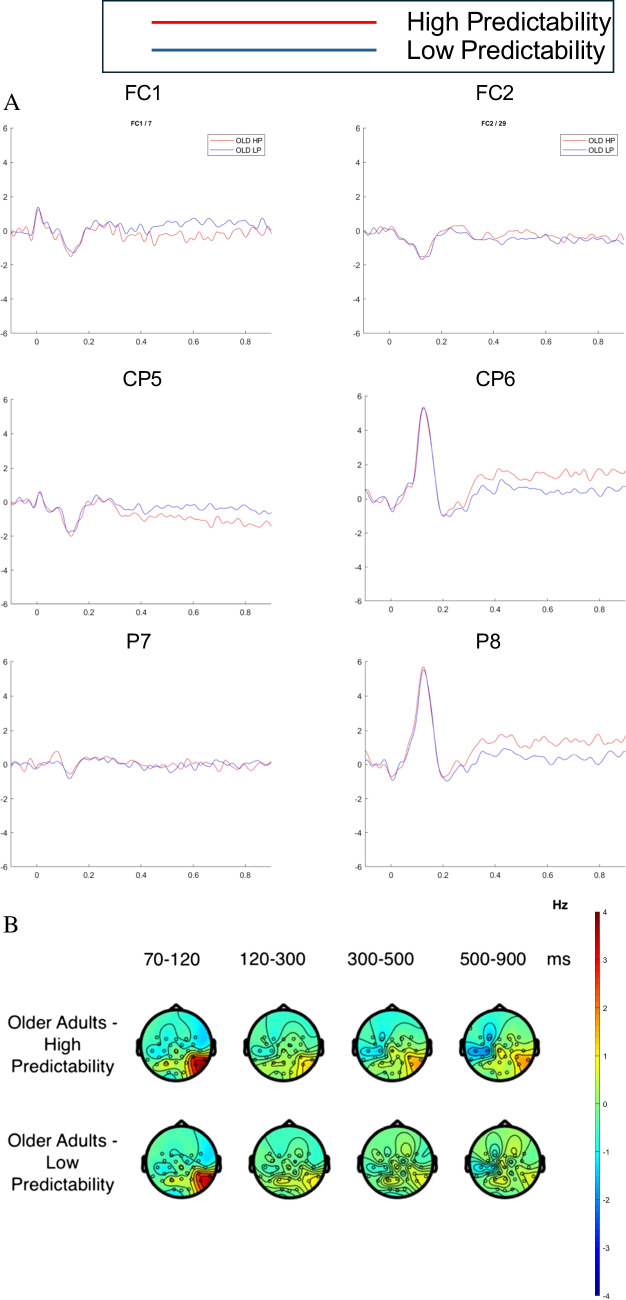
FRPs for the word-predictability effect for older adults. FRPs were time-locked to the onset of the initial first-pass fixation on the target word in each sentence. **A** Grand average FRPs associated with the word-predictability effect (LP versus HP) for older adults across six channel locations: left fronto-central (FC1), right fronto-central (FC2), left centro-parietal (CP5), right centro-parietal (CP6), left parietal (P7), and right parietal (P8). **B** Topographies of the average brain activity associated with the word -predictabilityeffect (LP versus HP) for older adults for four time windows: 70–120 ms, 120–300 ms, 300–500 ms, and 500–900 ms aftertheonsetof the initial first-pass fixation on the target word in each sentence. (Colour figure online)

Finally, we consider the interaction between age group and word predictability. The raster plot in Fig. 2C shows effects of word predictability for the older adults according to the cluster-based permutation tests (note that there were no corresponding significant effects for the young adults). Figures 5 and 6 depict FRPs for the young and older adults, respectively. To examine the interaction, we ran cluster-based permutation tests on comparison between the less predictable versus the highly predictable words for the older adults, and between the less predictable versus the highly predictable words for the young adults. These tests showed interaction effects. Cluster-based permutation tests performed separately for the two age groups revealed significant word-predictability effects for the older adults but not the young adults.

For the older adults, we observed significant clusters of positive voltage differences as well as clusters of negative voltage differences for less predictable compared to the highly predictable words. The raster plots (Fig. 2C) show that these activation clusters reflected an effect in the same direction over the same scalp region suggesting this might reflect a common aspect of processing. Specifically, we found a positive cluster over left central, fronto-central, and frontal brain regions, and a negative cluster largest over right temporo-parietal, parietal, and centro-parietal areas. The less predictable words elicited more negative amplitudes than highly predictable words over right temporo-parietal, parietal, and centro-parietal areas of the scalp (see Fig. 6). These topographies and waveforms might represent an N400-like effect; or, alternatively, they could reflect patterns of post-N400 late positivity/negativity. The topographies and waveforms show an effect over left central, fronto-central, and frontal brain regions such that the less predictable words elicited amplitudes with increased positivity compared the highly predictable words. It is unclear, however, whether this effect is the dipolar counterpart of the N400 effect or represents a late positivity effect for less predictable words (e.g., Federmeier et al., 2007; Thornhill & Van Petten, 2012), or even a late negativity for more predictable words (e.g., Wlotko et al., 2012).

Taken together, these interactive effects suggest that the older adults incurred a substantial cost in processing the less predictable words, and that their postlexical integration of these words with the prior sentence context was less efficient. One possibility is that older adults rely more on context to guide word identification, and so experience greater word identification difficulty and/or are slower to integrate words that are less predictable from context. By comparison, when words are integrated with sentential context more efficiently, as appears to be the case for young adults, effects of word predictability are attenuated. Another possibility is that older adults have greater difficulty recovering from a prediction error when an unexpected word is encountered in a highly constrained sentence context. The lack of predictability effects for young adults might initially appear surprising. However, attenuated word-predictability effects for young adults is consistent with effects in recent ERP studies using experimental paradigms that more closely approximate natural reading (e.g., Payne & Federmeier, 2017; Stites et al., 2017). Accordingly, the present findings are in line with the growing evidence that when parafoveal information is available and processing can proceed efficiently, the postlexical integration of words is accomplished at very different rates for older compared to younger adults.

## Discussion

With the present study, we used coregistered recordings of eye movements and EEG to gain insights into adult age differences in word-predictability effects in reading. This issue is important given the centrality of word predictability for the dominant cognitive models of reading (e.g., E-Z Reader, Reichle et al., 1998, 2003; SWIFT, Engbert et al., 2005; OB1-reader, Snell et al., 2018; Chinese Reading Model, Li & Pollatsek, 2020; SEAM, Rabe et al., 2023), and current theorizing about the more general role of linguistic prediction (e.g., Kuperberg & Jaeger, 2016; Pickering & Gambi, 2018). Moreover, as our research provides analyses of eye movements and brain activity in the same natural reading task, we anticipated that the findings might help to resolve a divergence in the characterization of age differences in context effects by researchers employing either standard eye-movement or ERP paradigms. Below we outline our findings and consider their implications for understanding how the use of context changes in older age.

### Eye-movement findings

In terms of the eye-movement behavior of our two groups of readers, sentence-level analyses confirmed that our experiment produced standard age differences, such that the older adults read sentences more slowly, by making more and longer fixations and more regressions, consistent with existing findings (see Zhang et al., 2022). We also found that the older adults had longer reading times for target words in sentences, in both measures of first-pass and later processing, and that the older readers were more likely to make a first-pass regression from the target words to re-read text earlier in the sentence. Again, these findings are consistent with previous eye-movement findings and suggest that older readers are generally slower to process words.

In terms of our manipulation of target-word predictability, we observed clear predictability effects for both age groups in word-skipping and fixation times. The word-skipping effect showed that both groups were more likely to skip high compared with the low predictability words, even though we made efforts to minimize target word-skipping. This demonstrates that a word’s predictability can influence the likelihood of it being identified parafoveally and a saccade being programmed to move past it (see Staub, 2015). At the same time, when the target word was fixated, both groups spent less time on high compared with low predictability words. This effect emerged in fixation time measures sensitive to early stages of a word’s processing (i.e., first-fixation durations and single-fixation durations), consistent with the skipping results for the two age groups. Crucially, we observed a differential effect of word predictably in later measures, such that the difference in reading times for highly predictable versus less predictable words was greater for the older adults. These later measures included regression-path reading times, which incorporate both the initial time spent processing a word (corresponding to gaze duration), as well as the duration of any fixations following a regression until the reader makes an eye movement to the right of the word (Liversedge et al., 1998). Accordingly, a larger word-predictability effect for the older readers in this measure additionally suggests that they looked back to words in the sentence context more when the target word was less predictable.

Note, however, that the degree to which older readers made a regression to re-read text prior to the target word was modest and did not differ much for high predictability words (mean = 13%) compared with low predictability words (mean = 17%). Moreover, neither did regression rates for the older adults differ much from those for the young adults (high-predictability words, mean = 4%; low-predictability words, mean = 6%). Furthermore, when the older readers made a regression, they did not spend much time re-reading the sentence context. For the older adults, regression-path times were 402 ms for low-predictability words (73 ms longer than gaze direction [GD] = 329 ms) and 343 ms for high-predictability words (51 ms longer than GD = 292 ms). The corresponding regression-path times for young adults were 298 ms for low-predictability words (32 ms longer than GD = 266 ms), and 253 ms (16 ms longer than GD = 237 ms) for high-predictability words.

We take these values to evidence several important points. First, both age groups made regressions from the target word on relatively few trials (i.e., 4–17% of trials). Therefore, on most trials and for both age groups, the initial processing of the target word was followed by a progressive saccade to its right. Furthermore, on that small proportion of trials when readers made a regression from the target word, they spent only a little time re-reading the sentence context. These points are important to hold in mind for when we discuss the FRP results. But also note that this does not affect our argument concerning the use of context by older readers. The effects we observed are consistent with older adults using context to a greater extent than young adults as they integrate words as part of a sentence’s meaning. However, this greater use of context did not cause the older readers to engage in substantially qualitatively different eye-movement behavior. Indeed, we consider that the present findings provide strong evidence that older adults have greater difficulty integrating words with context. Moreover, by using a well-powered design and not employing manipulations that might disrupt normal eye-movement behavior (e.g., parafoveal word misspelling), our findings reveal that, for alphabetic scripts like English, this occurs postlexically and relatively late during processing (cf. Choi et al., 2017). We note, however, that research in Chinese has obtained word-predictability effects earlier in processing, in gaze durations for words (Zhao et al., 2019). It is possible that specific processing requirements for different orthographies (in this case, an unspaced logographic script compared with the spaced alphabetic script) can modulate the time-course over which context effects are observed. That the present effect emerged relatively late during processing is also inconsistent with the view that older readers make greater use of context to predict upcoming words, as proposed within the “risky” reading hypothesis (Rayner et al., 2006). Such a mechanism would entail that age differences in context effects emerge early during processing, in fixation behavior associated with word identification, rather at later stages of sentence interpretation. Note also that this conclusion is in line with the meta-analysis reported by Zhang et al. (2022).

In sum, the eye-movement data reported here demonstrate that our participants were typical of young and older adult readers, that both groups use context to guide the early processing of words, but that older readers use this information to a greater extent, compared to young adults, as they integrate words to establish the meaning of the sentence. As noted earlier, this might be explained in terms of older readers having generally greater difficulty integrating less predictable words. Alternatively, it might provide evidence that older readers find it harder to recover from a prediction error, which occurs when they strongly predict a specific word but encounter a different, unpredicted, word. In this case, readers might need to suppress the incorrectly predicted word or revise their existing sentential representation to integrate the predicted word.

### Fixation-related potential findings

Turning to the FRP data, here we systematically explored effects pertaining to age, word predictability, and their interactive influence time locked to fixation onset on target words. First, we consider the main effects of age group; and here we note again that, to our knowledge, this is the first coregistration study to investigate effects of age on FRPs. Our results revealed robust effects of age. At a general level, observing these effects demonstrates that the coregistration paradigm is a valuable tool for advancing our understanding of aging effects in reading, and potentially broader aspects of visual cognition. Moreover, the paradigm may be useful in assessing differences in the time course of processing that occur with age. We obtained pervasive evidence of age differences in the FRP record. These effects were characterized by a rightward temporal shift in the waveform for the older relative to the younger adults. This shift to the right can be seen quite clearly in Figs. 2A and 3A and appears to represent a temporal lag in processing for the older adults during roughly the first 350 ms following fixation onset. Moreover, the period across which this lag occurs in the FRP maps quite closely onto the gaze duration time for target words (252 ms for young adults, and 311 ms for older adults). This corresponds to the period during which the target word underwent first-pass processing prior to an eye movement from this word.

The temporal delay in processing between older relative to young adults that we observed in the FRP results is consistent with proposals that cortical processing speed is slowed in older age (e.g., Salthouse, 1996), such that ERP components associated with different aspects of cognition, including word recognition (see Kutas & Federmeier, 2011), face perception (e.g., Daniel & Bentin, 2012), and cognitive control (e.g., Kropotov et al., 2016) have a delayed onset in older adults. As we noted above, older adults also have longer fixation times in these tasks. This raises the question of whether there is a link between fixation times on a stimulus and the onset latencies of specific FRP components in the record. Intuitively, this seems plausible, especially if decisions about where and when the eyes move are under cognitive control, which is a core theoretical assumption of many eye-movement models.

Previous research has considered the relationship between EEG and eye movements in the context of ERPs. For example, Sereno and colleagues explored possible relationships between ERP components and processing events that unfold during a fixation on a word, including word identification and saccade planning (Sereno & Rayner, 2000, 2003; Sereno et al., 1998). In a similar vein, Dambacher and Kliegl (2007), looked at the relationship between fixation durations and N400 amplitudes obtained from separate participant groups who read the same sentence stimuli, with the aim of establishing whether these were modulated by the same word properties and so, by implication, common mechanisms for word recognition. In line with this notion, the findings revealed a clear relationship between fixation durations on words and N400 amplitudes that could be accounted for in terms of the lexical frequency and predictability of the fixated word and predictability of the next (parafoveal) word. In both cases, explorations of these relationships were necessarily correlational, as the ERP was not time locked to specific fixations, only the onset of stimulus displays. Accordingly, an investigation of this question using FRPs seems promising precisely because FRPs are time locked to individual fixations and because their relationship can be investigated experimentally within the same individuals. Research using coregistration methods might therefore have the capacity to answer questions about this relationship, in terms of both associations and disassociations (Kutas & Federmeier, 2011; see also Burnsky et al., 2023; Kretzschmar et al., 2015).

Next, we consider FRP effects associated with word predictability. First, let us deal with the main effects of predictability that emerged in the FRP record. In the present case, the word-predictability effect was associated with differences around 300 ms (based on topography and waveforms) which might represent an N400-like component, with the amplitude attenuated for more predictable target words. N400 effects typically occur under experimental conditions where the semantic processing of linguistic and nonlinguistic stimuli is manipulated in respect of contextual fit. The N400 effect has been reported to correlate with the ease with which a word can be semantically integrated with the prior sentential context (e.g., Wlotko et al., 2010). Accordingly, as this effect is observed as a main effect, and obtained for both young and older adults in the present experiment, it appears that both groups experienced greater difficulty integrating the less predictable words with their understanding of the prior sentence context at this point in processing.

Crucially, the pattern of effects we observed in this component’s amplitudes contrast with those from ERP studies. ERP studies typically report interactive effects of age and word predictability in N400 amplitude, because of larger effects of word predictability for the young adults compared to the older adults, or an effect for young adults that is absent for older adults (e.g., Cameli & Phillips, 2000; Dave et al., 2018; DeLong et al, 2012; Federmeier & Kutas, 2005, 2019; Hamberger et al., 1995; la Roi et al., 2020; Payne & Federmeier, 2017; Wlotko & Federmeier, 2012a, b; Wlotko et al., 2012). That we obtained a main effect of word predictability for this component suggests both age groups experienced this effect.

An alternative characterization of the N400-like effect we observed is possible, however. It might instead reflect differences in early stages of processing associated with word identification, such as letter-to-whole-word form processing as per the N250 component described by Grainger and Holcomb (2009), or even the phonological and lexical processing of words as suggested by Degno et al. (2019b). Regardless of the precise interpretation, and whether the observed effect represents an early N400-like effect or an N250 component, it seems clear that the effect was driven by word predictability and that both young and older adults exhibited the effect. Again, it is noteworthy that the main effects for word predictability in the FRP record show good correspondence with the main effects in the eye-movement results (i.e., main effects of word predictability in first-fixation durations, single-fixation durations, and gaze durations). Accordingly, the behavioral and EEG evidence we obtained in the present experiment point to similar effects of word predictability on the early processing of words by both young and older adult groups during natural reading.

Next, let us consider the interactive effects of age and predictability that emerged. The interactive effects are striking and characterised by the word-predictability effect being more sustained for the older adults. These can be seen in Figs. 5 and 6, which depict waveforms for the young and older adults respectively, with the very pronounced effects for older adults alone shown in the raster plot (Fig. 2). Note that the pattern here is comparable to effects in regression-path and total reading times in the full EM dataset. These later activation effects suggest that the older readers engaged in more integrative processing for less compared to highly predictable words. That is, FRP effects were more pronounced and sustained for the older adults, suggesting increased cognitive processing relatively late in the FRP record when words were more difficult to interpret relative to the prior sentence context. Here we must be clear that we cannot distinguish between effects due to the less predictable words being more difficult to integrate or, alternatively, because words that were more predictable were easier to integrate. It also is possible that the effects are due to older readers having greater difficulty recovering from a prediction error on encountering an unpredicted word in a strongly constrained context. Finally, it is unclear whether these effects might relate to post-N400 positivity/negativity in some ERP studies (e.g., Wlotko et al., 2012), or earlier stages of processing associated with word identification (e.g., Grainger & Holcomb, 2009).

Earlier we noted that both age groups made relatively few regressions from the target words (4–17%), and most made a progressive saccade from the target word to inspect words to the right. It, therefore, seems unlikely that the interactive effects we observed in the later time windows are attributable to the two groups making qualitatively different patterns of eye movements (and thereby engaging in qualitatively different cognitive processing) after encountering the target word. Imagine, however, that they were associated with different patterns of eye movement. Say, for example, that after first encountering the target word, the older adults were substantially more likely to make a regression to re-read the sentence context, but that the young adults were much more likely to make a progressive eye movement, forward in the sentence, to read the next word. In this situation, it could be argued that the interactive effects we observe in FRPs are a consequence of the two groups of readers producing qualitatively different eye-movement behavior, which might be reflected in quite different patterns of processing. However, as the two groups exhibited quite similar eye-movement behavior on leaving the target word, it is unlikely that the processing effects we observe are driven by such basic differences in behavior (i.e., making a regressive as opposed to a progressive saccade). As such, it is reasonable to conclude that both groups were engaging in similar patterns of processing (i.e., attempting to integrate words with context), but that this progressed less effectively or efficiently for the older readers. While this interpretation seems credible, further work is needed to more fully understand the nature of the processing differences between young and older adults.

We can also speculate that the late negativity we observed may be like late effects in some ERP studies as a post-N400 positive deflection around 600–900 ms following stimulus onset. One possibility is that this pattern reflects a processing cost for incorrect predictions, which is proposed to elicit late positivity (see Wlotko & Federmeier, 2012a, b; Wlotko et al., 2010, 2012). ERP findings are somewhat mixed, however, showing such an effect either selectively for young adults (Wlotko et al., 2012), or for both young and older adults (Dave et al., 2018; DeLong et al., 2012). Moreover, the effect we observed was for older adults only; therefore, if this did represent a processing cost for prediction error, our findings might imply that this cost was not incurred by the young adults. Another possibility is that our effect corresponds to late negativity observed by Wlotko et al. (2012) only for older readers and attributed to these readers engaging in late processes of contextual integration to compensate for a reduced ability to use contextual information predictively. However, as we observed a predictability effect for both age groups early in the FRP, it seems unlikely that the effect represents a delay in the initiation of integrative processes by our older readers. Moreover, it is striking just how pervasive this pattern of effects is for the older readers. Whether this represents a processing cost for prediction error, or protracted difficulty integrating words that are not strongly predicted, it seems likely that such processing occurs frequently during reading for cognitively unimpaired older adults like the participants in the present study. This raises the question of just how much this disrupts their processing of written information in daily life. Clearly it also will be valuable to establish what aspects of cognitive aging are associated with this extensive pattern of potential processing difficulty.

In sum, by employing coregistration methods, the present research provides complementary evidence from eye movements and FRPs that older readers make greater use of context to process words in natural reading. In presenting these findings, we note the close correspondence of effects observed in eye movements and FRP waveforms, as well likely correspondences with effects in previous ERP research using less natural reading paradigms. What seems especially clear is that the use of coregistration of eye movements and EEG has the capacity to advance our understanding of aging effects in natural reading.

## Supplementary information

Below is the link to the electronic supplementary material.

Supplementary file1 (DOCX 649 KB)

## Data Availability

The data and materials for all experiments are available online via the OSF data repository: https://osf.io/aq7g6/?view_only=84b8cb03449f4f04979d82d411aaab7b. The reported experiment was not preregistered.

## Footnotes

1 In addition to conducting analyses across a single time window from 70–900-ms posttarget word-fixation onset, we performed additional analyses across a series of predefined time windows (70-120 ms, 120–300 ms, 300–500 ms, and 500–900 ms). These were selected as windows within which key FRP components (e.g., P1, N1, N400, P600, late frontal positivity/negativity) might be observed. We include these analyses (which are also available from in our OSF files) as supplementary information so that interested readers may compare our results and those from studies that focused on specific ERP/FRP components. We note that the results we obtained across these predefined windows resemble those we report for the single time window.
