# Correction to: Aging and word predictability during reading: Evidence from eye movements and fixation-related potentials

**DOI:** 10.3758/s13414-025-03070-1

**Published:** 2025-04-28

**Authors:** Ascensión Pagán, Federica Degno, Sara V. Milledge, Richard D. Kirkden, Sarah J. White, Simon P. Liversedge, Kevin B. Paterson

**Affiliations:** 1 School of Psychology and Vision Sciences, George Davies Centre for Medicine, University of Leicester, University Road, Leicester, LE1 7RH UK; 2 Department of Psychology, Bournemouth University, Poole, UK; 3 School of Psychology, University of Central Lancashire, Preston, UK

**Correction to: Attention, Perception, & Psychophysics (2025) 87:50–75**

10.3758/s13414-024-02981-9

The scale accompanying the topographies in Figures 3B, 4B, 5B, and 6B showed an incorrect voltage range. This should range between +/- 4 volts and not +/- 1 volts as shown in the originally published article.

The original article has been corrected.

