# Supplementary material for: Aging and word predictability during reading: Evidence from eye movements and fixation-related potentials
Source: Atten Percept Psychophys. 2025 Jan 28;87(1):50–75. doi: 10.3758/s13414-024-02981-9 (PMC11845442; doi:10.3758/s13414-024-02981-9)
Supplement: Supplementary file 1 — Supplementary file1 (DOCX 649 KB) [file 13414_2024_2981_MOESM1_ESM.docx]

**Supplementary analysis on FRP data**

We conducted additional cluster-based permutations tests to analyse the effect of group, word predictability, and their interaction over four time windows (i.e., 70-120ms, 120-300ms, 300-500ms and 500-900ms). We selected these time windows to examine key FRP components associated with processing in natural reading (e.g., P1, N1, N400, P600, late frontal positivity/negativity). These additional analyses allow for the interested reader to make comparisons between the FRP results from the present experiment and results from other ERP or FRP studies that have chosen to focus their analyses on specific waveform components. Below, we include a summary of statistical results for the analyses for these four time-windows in Table S1. Figure S1 uses raster diagrams to show the pattern of FRP effects across all channels for each time-window.

Table S1. Summary of Statistical Effects in Fixation-Related Potentials across Four Pre-Defined Time-Windows.

| Effects | Cluster | Time Windows | | | | | | | | | | |
| --- | --- | --- | --- | --- | --- | --- | --- | --- | --- | --- | --- | --- |
|  |  | 70-120 ms | |  | 120-300 ms | |  | 300-500 ms | |  | 500-900 ms | |
|  |  | N | p |  | N | p |  | N | p |  | N | p |
| **Main Effects** |  |  |  |  |  |  |  |  |  |  |  |  |
| Group (Older-Young) | Positive | 1 | <.003* |  | 2 | <.001* |  | 1 | <.022* |  | 0 | - |
|  | Negative | 1 | <.012* |  | 2 | <.001* |  | 1 | >.114 |  | 0 | - |
| Predictability (Low-High) | Positive | 0 | - |  | 0 | - |  | 3 | >.075 |  | 4 | > .040 |
|  | Negative | 0 | - |  | 1 | <.008* |  | 1 | <.008* |  | 3 | >.145 |
| Group x Predictability | Positive | 0 | - |  | 0 | - |  | 1 | <.010* |  | 2 | <.019* |
|  | Negative | 0 | - |  | 0 | - |  | 2 | <.023* |  | 4 | < .015 |
| **Contrasts** | |  |  |  |  |  |  |  |  |  |  |  |
| Young | Positive | NA | NA |  | NA | NA |  | 1 | >.049 |  | 1 | >.110 |
| (Low vs High Predictability) |  |  |  |  |  |  |  |  |  |  |  |  |
|  | Negative | NA | NA |  | NA | NA |  | 1 | >.093 |  | 0 | - |
| Older | Positive | NA | NA |  | NA | NA |  | 2 | <.007* |  | 4 | <.024* |
| (Low vs High Predictability) |  |  |  |  |  |  |  |  |  |  |  |  |
|  | Negative | NA | NA |  | NA | NA |  | 3 | <.020* |  | 2 | <.003* |

*Note*. Significance is when p values are < .25. Asterisks show clusters that are significant. NA: no cluster-based permutation test was conducted. Asterisks show clusters that are significant.

Figure S1. Raster Diagrams of Significant FRP Effects Across the Four Pre-Defined Time-Windows using Cluster-Based Permutation Tests.


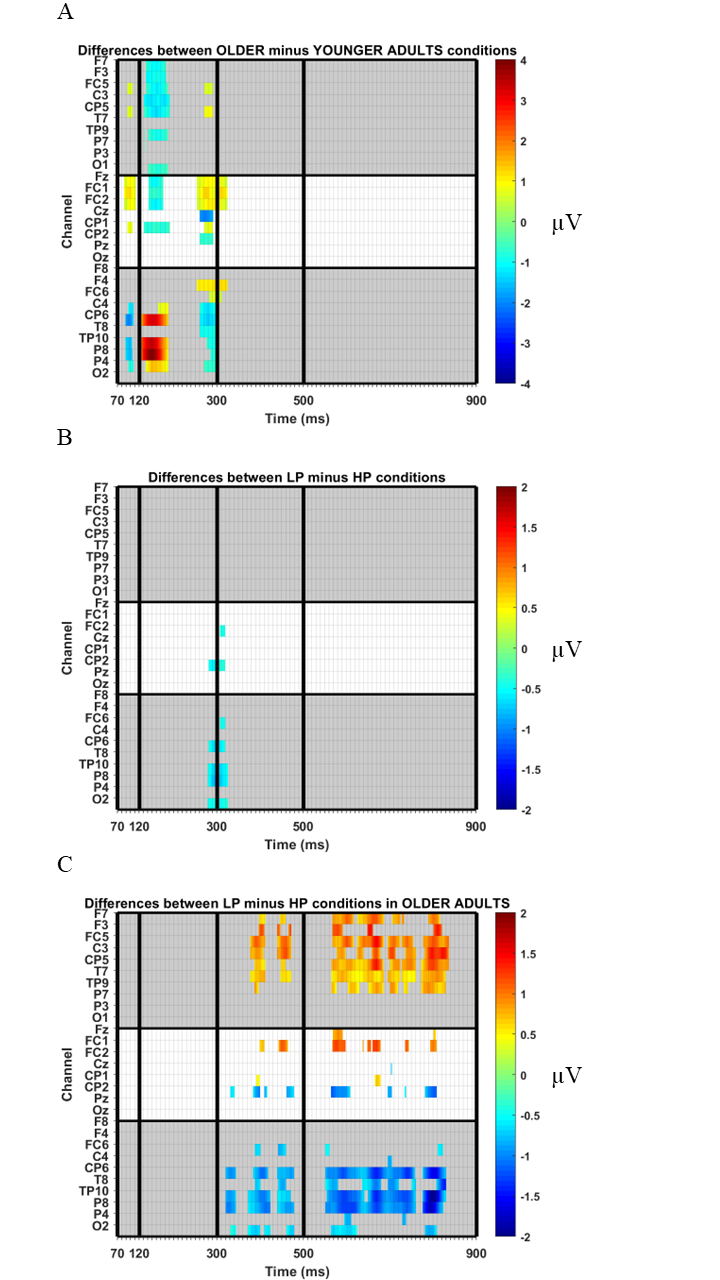


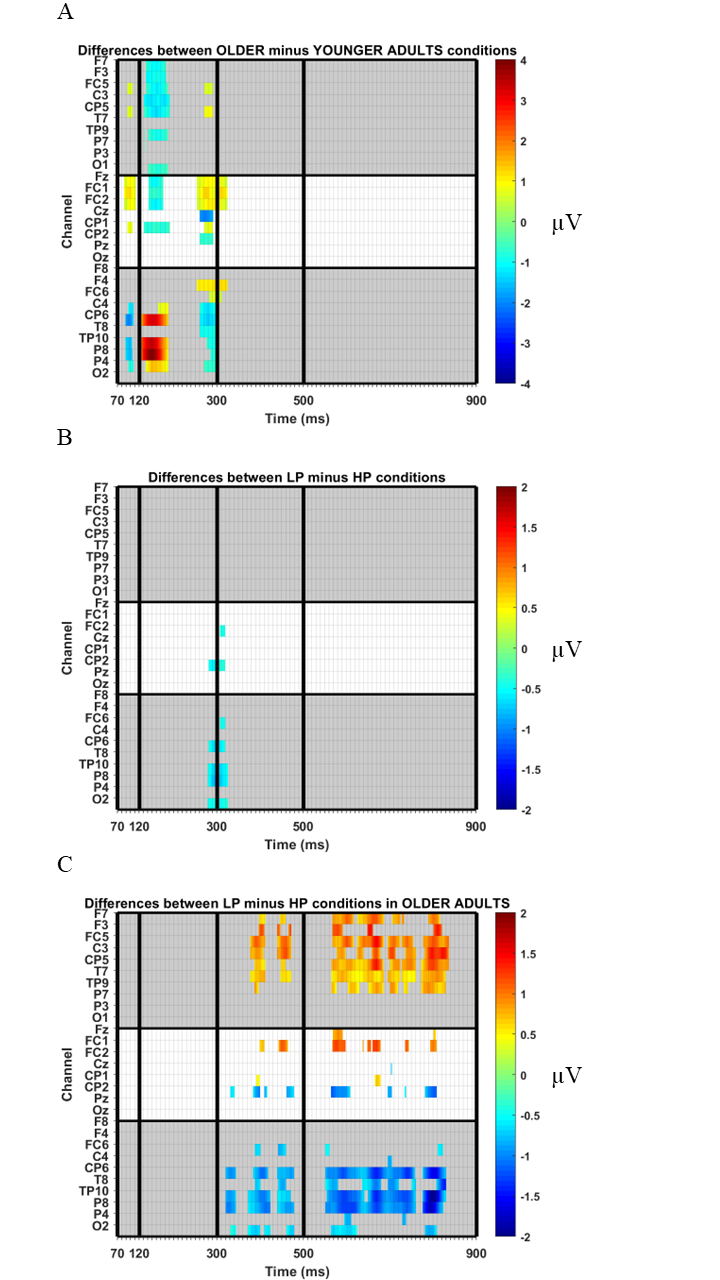


Panel A shows FRP differences between older and younger adults

Panel B shows FRP differences between low predictability (LP) and high predictability (HP) target words.

Panel C shows FRP differences between low predictability (LP) and high predictability (HP) target words for older adults. Note that FRP difference for LP and HP for the younger adults are not included as these showed no significant effects.

Red and blue rectangles indicate the channel/time point in which one levels of a condition (i.e., older versus younger adults, low predictability [LP] versus high predictability [HP]) is significantly more positive or negative than the second condition.

Channels are displayed on the y-axis and organized somewhat topographically. Channels within the left hemisphere of the scalp are shown on the top grey rectangle. Midline electrodes are displayed in the middle (unfilled) section, and channels within the right hemisphere are shown on the figure’s bottom grey rectangle. The time from the onset of a fixation on the target words is displayed on the x-axis. Vertical black lines demarcate the time windows use for cluster-based permutation tests: 70–120 ms, 120–300 ms, 300–500 ms, and 500–900 ms following fixation onset.
